# CART—a chemical annotation retrieval toolkit

**DOI:** 10.1093/bioinformatics/btw233

**Published:** 2016-06-02

**Authors:** Samy Deghou, Georg Zeller, Murat Iskar, Marja Driessen, Mercedes Castillo, Vera van Noort, Peer Bork

**Affiliations:** ^1^Structural and Computational Biology Unit, European Molecular Biology Laboratory, Heidelberg, Germany; ^2^Centre of Microbial and Plant Genetics, KU Leuven, Leuven, Belgium; ^3^Molecular Medicine Partnership Unit, University of Heidelberg and European Molecular Biology Laboratory, Heidelberg, Germany; ^4^Max Delbrück Centre for Molecular Medicine, Berlin, Germany; ^5^Department of Bioinformatics, Biocenter, University of Würzburg, Würzburg, Germany

## Abstract

**Motivation**: Data on bioactivities of drug-like chemicals are rapidly accumulating in public repositories, creating new opportunities for research in computational systems pharmacology. However, integrative analysis of these data sets is difficult due to prevailing ambiguity between chemical names and identifiers and a lack of cross-references between databases.

**Results**: To address this challenge, we have developed CART, a Chemical Annotation Retrieval Toolkit. As a key functionality, it matches an input list of chemical names into a comprehensive reference space to assign unambiguous chemical identifiers. In this unified space, bioactivity annotations can be easily retrieved from databases covering a wide variety of chemical effects on biological systems. Subsequently, CART can determine annotations enriched in the input set of chemicals and display these in tabular format and interactive network visualizations, thereby facilitating integrative analysis of chemical bioactivity data.

**Availability and Implementation**: CART is available as a Galaxy web service (cart.embl.de). Source code and an easy-to-install command line tool can also be obtained from the web site.

**Contact**: bork@embl.de

**Supplementary information:** Supplementary data are available at *Bioinformatics* online.

## 1 Introduction

Understanding the effects of chemicals, in particular small organic molecules, on biological systems is fundamental to research in pharmacology, toxicology, chemical biology and related fields. Bioactivities of chemicals can be investigated at various scales analyzing drug-associated readouts, such as protein interactions, cellular phenotypes, toxicity or side effects (Iskar *et al.*, 2012). Owing to the development of high-throughput screening technologies, bioactivity data for large chemical libraries has rapidly accumulated in recent years and is increasingly becoming available in public repositories (see Table 1). While this has created tremendous opportunities for research that aims to integrate these heterogeneous data sets in order to gain a better systemic understanding of chemical effects, in practice such efforts are severely impeded by disparities in data representation. In particular, unambiguous identification of chemicals across databases can be difficult, because a myriad of synonyms and trade names exist for many chemicals, and even controlled nomenclature and structural descriptions are sometimes ambiguous, similar to the problem of mapping between various gene, transcript and protein nomenclatures, now overcome by many bioinformatics tools (Huang *et al.*, 2009, among others). To address the persisting need in chemoinformatics, we here present CART, a Chemical Annotation Retrieval Toolkit. In solving the chemical name-matching problem, CART aims at integrating bioactivity annotations across various databases to provide functional annotation and enrichment analysis for chemicals. Thereby CART can identify coherent functional themes, analogous to gene ontology annotation tools, such as DAVID (Huang *et al.*, 2009). This makes CART useful, e.g. for the automatic characterization of hits derived from chemical screens (Rihel *et al.*, 2010, for instance). Also in other contexts, annotating chemicals with various biological effects is becoming an important task, which has so far largely required expert manual annotation, but can be greatly simplified by CART.

**Table 1.** Chemical bioactivity databases available through CART

Bioactivity	Database	Size^a^	References
Molecular target	STITCH	221 724 / 9015	stitch.embl.de
	TTD	11 340 / 1120	bidd.nus.edu.sg/group/cjttd
	DrugBank	853 / 147	www.drugbank.ca
Gene interactions	CTD	6334 / 8346	ctdbase.org
Metabolization	DrugBank	396 / 64	www.drugbank.ca
Therapeutic class.	ChEMBL	1118 / 1538	www.ebi.ac.uk/chembl/ftc
	ATC	2515 / 924	www.whocc.no/atc
Drug side effects	SIDER	1309 / 4130	sider.embl.de
Toxicity	DrugMatrix	742 / 22	ntp.niehs.nih.gov/drugmatrix

^a^Annotated chemicals/annotation terms, see Supplementary Figure S3 and Supplementary Material S2.

## 2 Approach

**Fig. 1.**
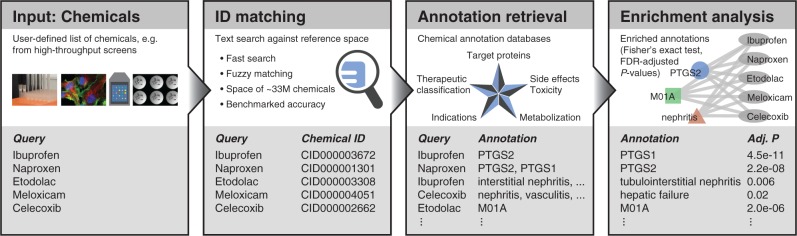
Typical CART workflow including chemical name matching, annotation retrieval and enrichment analysis. The lower panels contain a toy example of non-steroidal anti-inflammatory (NSAID) compounds and show excerpts of how these are matched and annotated by CART, the rightmost panel displays a (partial) enrichment network; PTGS, prostaglandin-endoperoxide synthase targets; M01A, ATC code for NSAIDs, Adj. *P*, FDR-corrected *P*-value, nephritis and vasculitis are NSAID-associated side effects. See Supplementary Material S3 and Supplementary Figure S4 for an application of CART to hits from a drug screen.

The first component of CART consists of matching user-provided chemical names to a comprehensive dictionary of synonyms, serving as a reference space for disambiguation to unique chemical identifiers (Fig. 1). To improve matching sensitivity over exact synonym look-up, we additionally implemented an approximate text matching method based on the Apache Lucene search engine (http://lucene.apache.org/) and heuristics such as the conversion between salt (e.g. salicylate) and acid form (salicylic acid, see Supplementary Material S1 for details). CART also offers the possibility to match structural chemical identifiers, SMILES and InChI keys, via exact string matching. Taken together, these search capabilities go beyond what existing tools, such as e.g. CTD (Davis *et al.*, 2014), currently offer (see Supplementary Table S1).

Mapping to CART's chemical reference space facilitates subsequent retrieval of bioactivity annotations (Table 1, Supplementary Material S2). This allows for easy, multi-facetted annotation of chemical libraries, synonym retrieval, which is useful e.g. for text mining, and the identification of bioactivities that are enriched in the user-provided input. Statistical significance for these enrichments is established using Fisher’s exact test with FDR correction for multiple testing.

In a typical use case, users may want to subject a set of hits resulting from a high-throughput chemical screen to CART analysis. After name matching, the enrichment analysis can be done relative to a user-specified background, in this case the library of all chemicals probed in the screen. Enriched annotations are subsequently retrieved from databases describing chemical effects at various scales, including molecular targets, metabolizing enzymes, functional classifications, indication areas and side effects (Table 1, Supplementary Material S2). The results are visualized as a network linking the input set of chemicals to enriched annotations (Fig. 1, Supplementary Material S3, Supplementary Figure S4). Implemented in Cytoscape.js (Franz *et al.*, 2015), this network can be interactively explored.

The Galaxy (Goecks *et al.*, 2010) front-end of CART enables users to combine individual modules into new workflows, allowing for easy customization and extension of the standard use case described above. Galaxy moreover facilitates reproducibility due to its history and sharing functionalities (Goecks *et al.*, 2010).

## 2 Results

CART uses a comprehensive chemical reference space of about 98.8 million names and synonyms and 68.3 million InChIKeys that are disambiguated to 37.7 million chemical identifiers based on information from the STITCH database version 4.0 (Kuhn *et al.*, 2014). Matching user-provided chemical names into this reference space is very fast, e.g. processing 1,000 chemicals takes  <40 s (Supplementary Figure S1), allowing integrative analyses at a large scale. This is becoming crucial due to the data deluge of publicly available chemical bioactivity data (Wang *et al.*, 2012).

We benchmarked the accuracy of CART's (approximate) name matching algorithm using four datasets, for which a mapping to STITCH or PubChem identifiers already existed so that they could serve as a gold standard. We found CART’s sensitivity to range between 92 and 100% on these benchmarks, while precision ranged between 79 and 98% (Supplementary Figure S2). As an additional means of ensuring high analysis standards, CART enables the user to interactively curate the automatic name matching results before proceeding further.

Owing to its unified reference chemical space, CART offers seamless integration of user-provided data with a number of databases containing functional annotations of chemicals at various scales (Table 1). These databases vary in scope, as the number of annotated chemicals ranges from >220 000 compounds with known protein interactions (Kuhn *et al.*, 2014; Qin *et al.*, 2014) to a few hundred drugs for which therapeutic classification, metabolization and toxicity information (Croset *et al.*, 2014; Kuhn *et al.*, 2015; Law *et al.*, 2014) is publicly available (Supplementary Figure S3). However, for a set of 1,120 well-characterized chemicals, annotations from ≥5 databases are provided (Supplementary Figure S3). CART’s annotation and enrichment functionality is demonstrated on drug sets previously defined in a study by Rihel *et al.* (2010) that screened chemicals for behavioural effects on zebrafish larvae (Supplementary Material S3 and Supplementary Figure S4). It revealed coherent themes of drug bioactivities, which could otherwise only be discovered by expert manual annotations (as done in Rihel *et al.*, 2010).

In summary, CART implements a fast and accurate approach for matching chemical names to a comprehensive chemical universe. This facilitates the retrieval of enriched annotations from various databases describing chemical effects on biological systems (Table 1) and their exploration in an interactive network view. CART thus makes integrative analysis of chemical bioactivity data easy even for non-specialists.

## Supplementary Material

Supplementary Data
