# Supplementary material for: CART—a chemical annotation retrieval toolkit
Source: Bioinformatics. 2016 Jun 2;32(18):2869–71. doi: 10.1093/bioinformatics/btw233 (PMC5018367; doi:10.1093/bioinformatics/btw233)
Supplement: Supplementary Data [file supp_32_18_2869__index.html]

CART—a chemical annotation retrieval toolkit — CART—a chemical annotation retrieval toolkit — Supplementary Data 

# CART—a chemical annotation retrieval toolkit

## Supplementary Data

files

- Supplementary Data - docx file
