# Supplementary material for: CART—a chemical annotation retrieval toolkit
Source: Bioinformatics. 2016 Jun 2;32(18):2869–71. doi: 10.1093/bioinformatics/btw233 (PMC5018367; doi:10.1093/bioinformatics/btw233)
Supplement: Supplementary Data [file supp_btw233_CART-supp-info.docx]

Supplementary Information

for

CART – a chemical annotation retrieval toolkit

Contents

1. Supplementary Figure 1. Benchmark of CART’s name matching runtime 2

2. Supplementary Figure 2. Benchmark of CART’s name matching accuracy 3

3. Supplementary Figure 3. Annotation database coverage 4

4. Supplementary Figure 4. Network visualization of enriched bioactivities in drug Cluster 10 of Rihel et al. (2010) 5

5. Supplementary Note 1. Details on CART’s name matching functionality 6

6. Supplementary Note 2. Chemical annotation databases integrated into CART 7

7. Supplementary Note 3. Functional enrichment analysis of drug sets using CART: Re-analysis of a zebrafish behavioral drug screen 8

8. Supplementary Table 1. Comparison with the existing tools 9


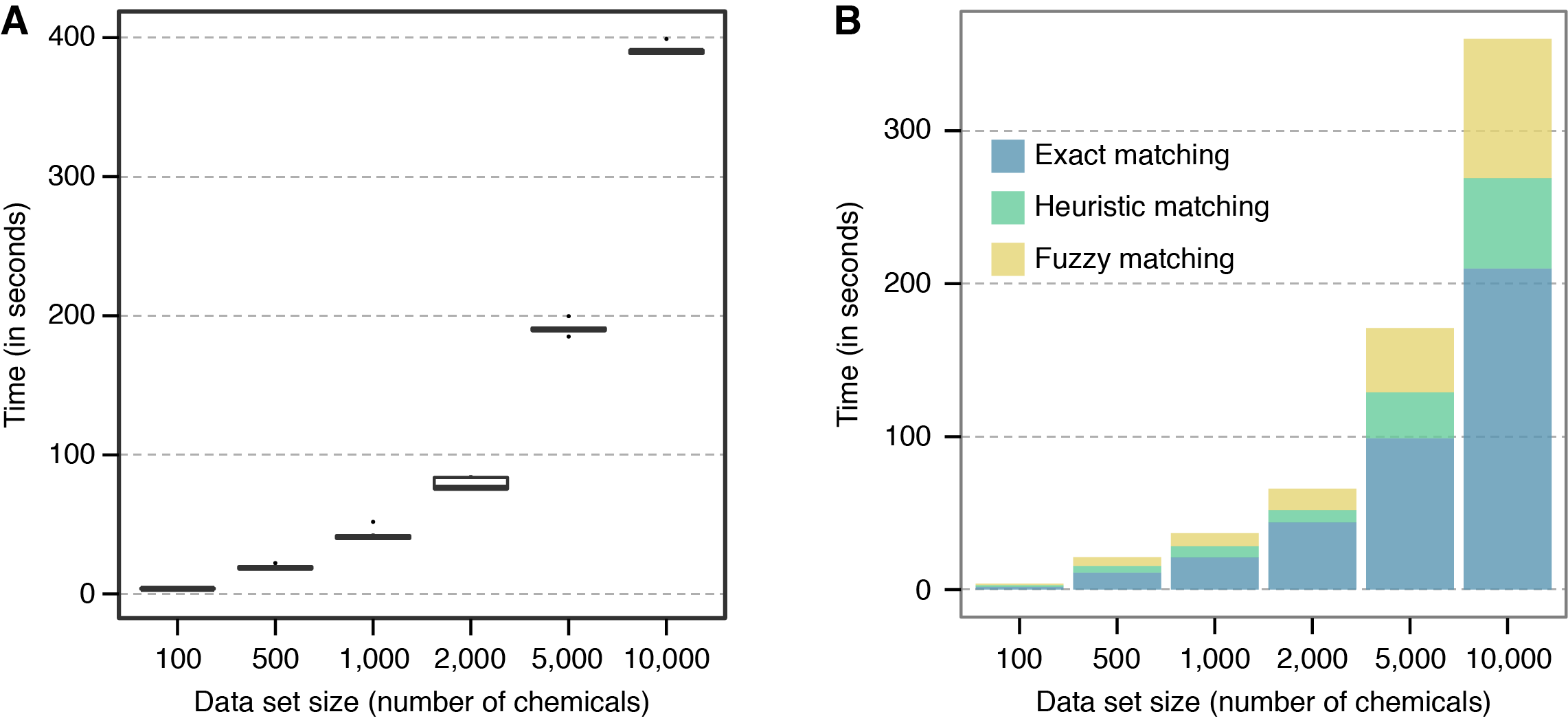


**Supplementary Figure 1. Benchmark of CART’s name matching runtime.**

CART uses text matching to map user-defined chemical names to a synonym index. CART’s synonym index was constructed using the STITCH chemicals and chemical aliases files (version 4.0) from which it incorporates all ‘flat’ compound IDs, which merge stereo-isomers (Kuhn et al. 2014). The name matching proceeds in three steps. First it tries to find an exact match between the user input and compound synonyms. Where no exact match is found, CART attempts fuzzy (approximate) matching and also applies some heuristic conversion rules, e.g., by removing low information suffices such as e.g. “hcl” (details can be found in Supplementary Note 1).

To benchmark CART’s name matching run times, we generated data sets of increasing size by randomly selecting compound names from PubChem. These were then processed with CART’s name matching module (commandline version) on a Macintosh 2.8Ghz Intel Core i5 with 8GB of RAM to record run times. (A) Boxplots for 10 resampled runs for each data set size. (B) Runtimes broken down by the different matching steps for one run per data set size.


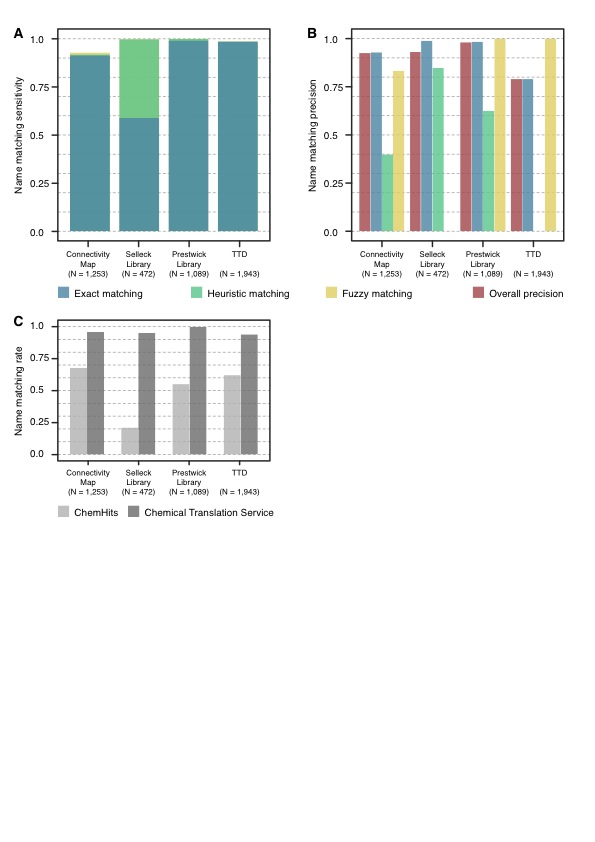


**Supplementary Figure 2. Benchmark of CART’s name matching accuracy.**

As CART’s compound identification is based on approximate text matching rather than comparison of chemical structures, we sought to assess errors incurred in the matching process, which might confound downstream analysis. For this we evaluated CART’s name matching on four data sets for which an independent (manually curated) mapping of chemicals to STITCH IDs (or PubChem IDs, which can be trivially converted to STITCH IDs) existed. (A) Sensitivity, defined as the fraction of input chemicals that could be matched to STITCH IDs for the four benchmark data sets (size indicated in brackets) colored by matching step; as these are combined in a cascaded approach (subsequent matching steps only applied to the unmatched remainder of the previous matching step), total sensitivity can be calculated in an additive manner. (B) Precision, defined as the fraction of correct matches among all matched chemical names, for the overall combined matching as well as individual approaches. Except for the TTD benchmark set overall precision was found to exceed 90%. (C) Match rates for chemical name searches performed with ChemHits (sabio.h-its.org/chemHits) and Chemical Translation Service (cts.fiehnlab.ucdavis.edu). Note that correctness of matches was not assessed (as the returned identifiers are not directly comparable or independently mappable to the ones that CART uses), only the fraction of input chemicals returning any match is shown. While ChemHits searches are as fast as CART’s, querying the Chemical Translation Service via its RESTful API took much longer (>2 sec / chemical, i.e. 50 - 80 mins for each of the data sets shown).

**Supplementary Figure 3. Annotation database coverage.**

(A) Number of chemicals (unique STITCH ‘flat’ IDs with stereo-isomers merged) for which at least one bioactivity annotation exists in the respective database. (B) Number of chemicals shared between databases. For example, there are 2,789 chemicals that have at least one bioactivity annotation in three or more databases.

**Supplementary Figure 4. Network visualization of enriched bioactivities in drug Cluster 10 of Rihel et al., 2010.**

In this network, chemicals are represented by gray rounded rectangles, protein targets (STITCH, TTD, DrugBank) by blue circles and functional classifications (FTC) and drug indications (ATC code) with green squares. Edges link input chemicals to their (significantly enriched) annotations. The network clearly shows the histamine receptor H1 to be the primary target of most drugs from Cluster 10 consistent with enriched ATC codes R06(A) and similar FTC codes for “Anti-histamine receptor activity agent”. (CYP3A5: cytochrome P450, family 3, subfamily A, polypeptide 5, CYP3A7: cytochrome P450, family 3, subfamily A, polypeptide 7)

**5. Supplementary Note 1. Details on CART’s chemical matching functionality.**

There are 3 input types that CART can match against its internal chemical universe of synonyms:

1. **Chemical names**: Each chemical name is matched against its chemical synonym universe using the Apache Lucene text search engine API solr using the three matching algorithms detailed below.
2. **InChIKeys**: InChIKeys are matched against a database of InChIKeys corresponding to CART’s internal universe of chemicals using exact string search.
3. **SMILES**: SMILES chemical descriptors are first transformed into InChIKeys and these are in turn matched to CART’s InChIKey database.

In brief, CART’s name matching proceeds in a cascade of three approaches, in which only unmatched input chemicals from the previous step are subjected to the subsequent matching approach.

1. **Exact matching:** For the exact match Lucene performs a simple string comparison between the input chemical names and every chemical name from the chemical synonym universe. It returns a match if and only if the compared names are identical. Chemicals without matches will then be subjected to the next step.
2. **Heuristic Matching:** For the heuristic match the following low information terms are trimmed from the input chemical names: “ hcl”, “hydrochloride”, “dihydrohloride”, “chlorhydrate”, “salt”, “potassium”, “dihydrate”, “acid”, “oxid”, “chloride”, for instance “tramadol hydrochloride” becomes “tramadol”. Lucene then matches the modified chemical name (“tramadol”) against all chemical synonyms, this time by allowing non-perfect string matches (using the fuzzy matching explained below). Chemicals without matches will then be matched fuzzily but without modification (in this case “tramadol hydrochloride”).
3. **Fuzzy Matching:** For this, Lucene compares the input chemical names to its synonym database allowing non-perfect string matches. This can be useful when the input chemical name contains typos (for instance “acetaminiphen” will be matched to “acetaminophen”). To ensure high specificity for these approximate text matches, we compute a match score S (value between 0 and 1) for each pair of input chemical and CART synonym. If S exceeds a threshold t (set by the user, default value is 0.9), then this match is returned to the user. S is computed as follows:
   S = 1 – (D(input, synonym) / max(length(input), length(synonym))
   where D is the Levenshtein distance, also known as edit distance. For matches with the same score, one is returned at random.
   Examples:
   S(“ibuprofen”, “ibuprofen”) = 1
   S(“acetaminiphen”, “acetaminophen”) = 0.93

**6. Supplementary Note 2. Chemical annotation databases integrated into CART**

We integrated various resources on bioactivities of chemicals into CART’s annotation retrieval and enrichment calculation modules. In total, we included information from seven distinct databases:

1. Protein targets from the Search Tool for InteractTions of CHemicals (STITCH ver. 4.0)(Kuhn *et al.*, 2014),
2. Therapeutic targets from the Therapeutic Target Database (TTD ver. 4.3.02)(Zhu *et al.*, 2012),
3. Anatomical Therapeutic Chemical classification system (ATC code available from STITCH ver. 4 “downloadable files”),
4. The Functional Therapeutic Classification system from ChEMBL (ChEMBL: FTC ver. 1)(Croset *et al.*, 2014),
5. Drug side effects from the Side Effect Resource (SIDER ver. 4.1)(Kuhn *et al.*, 2015),
6. Molecular targets and metabolization information from DrugBank (ver. 4.1)(Law *et al.*, 2014),
7. Manually curated chemical-gene interactions with a focus on toxic agents as made available by The Comparative Toxicogenomics Database (CTD, downloaded in Feb 2016) (Davis *et al.*, 2015),
8. Toxicity annotations of compounds originally extracted from literature using text-mining as provided by the DrugMatrix database (file last modified on 5 April 2012)

Specifically, from the STITCH resource we retrieved high-confidence chemical-protein interactions for Homo sapiens, which had at least a (medium) evidence score of 0.4 from experimental and/or database sources, thus excluding interactions that are only based on text mining. ATC codes were downloaded from the STITCH resource. Drug side effects were extracted from the SIDER database using STITCH IDs as provided. Chemical-gene interactions were retrieved from the CTD website (<http://ctdbase.org/downloads/#cg>). Also from CTD, we only retained the toxicogenomic interactions reported for the proteins from Homo sapiens. If PubChem/STITCH IDs were not provided as part of the database, chemical names were matched to the flat chemical IDs (stereo-isomers merged) in the STITCH chemical space. Chemical names from CTD were matched to the corresponding STITCH CIDs using CART’s name matching capabilities. Therapeutic drug targets were downloaded from DrugBank and TTD using the provided cross-references to PubChem and mapped to our chemical reference space using CART’s name matching routines. Similarly, toxicity annotations were parsed from DrugMatrix’s literature annotations using CART. For DrugBank, CTD and DrugMatrix databases, chemical IDs were additionally manually annotated to ensure correctness and completeness. Target information from Drugbank was divided into two separate parts depending on whether the target was annotated as mediating the therapeutic effect of the compound or its metabolization (as e.g., for cytochromes). We show the total number of distinct chemicals per database in Supplementary Figure 3A. Additionally, Supplementary Figure 3B summarizes the number of chemicals shared between databases, e.g. 1,466 chemicals have annotation(s) in four or more of the databases.

**7. Supplementary Note 3. Functional enrichment analysis of drug sets using CART: Re-analysis of a zebrafish behavioral drug screen (Rihel et al., 2010)**

To illustrate functional enrichment analysis with CART, we applied it to clusters of drugs identified previously by Rihel et al., 2010 based on similarity of behavioral effects on larval zebrafish. In this screen, a broad range of psychotropic drugs was clustered into 24 drug clusters (see Supplementary Figure 3 of Rihel et al. 2010). For our enrichment analysis, we used all screened drugs as ʻbackgroundʼ and ran CART on each of the drug clusters to identify enriched i) molecular targets (from STITCH, TTD and DrugBank), ii) metabolizing enzymes (from DrugBank), iii) functional classifications (from ChEMBL), iv) drug indication areas (from ATC), v) side effects (from SIDER), vi) and toxicity annotations (from DrugMatrix). All the enrichment results (FDR corrected P value < 0.05) are provided as publicly accessible Galaxy histories (http://cart.embl.de/history/list_published). CART analysis identified a wide variety of enriched drug targets and biological processes. In ideal cases, it revealed coherent themes of drug bioactivities in these clusters, which could otherwise only be discovered by expert manual annotations (as done in Rihel et al., 2010)(Rihel *et al.*, 2010). In the following we discuss three such examples out of the 24 that are accessible online (http://cart.embl.de/history/list_published). Taken together, these cases demonstrate how CART can be useful to automatically characterize hit sets derived from high-throughput drug screening.

As a first example, we analyzed Cluster 17 that was originally associated with a sedative phenotype in the zebrafish screen. CART identified the “α-2 adrenergic receptor” (ENSP00000280155, FDR-corrected P value 0.017) as an enriched target and “Pro-alpha-2C adrenergic receptor binding agentʼs (FTC_P0031696, FDR-corrected P value 5.26E-5) as an enriched ChEMBL therapeutic classification, consistent with what Rihel et al. originally reported based on manual annotation.

As a second example, we applied CART to Cluster 2. In the original publication, Rihel et al. highlighted an unexpected association of anti-inflammatory agents in this cluster with the increased waking activity of zebrafish larvae during the day. Consistent with this, CART analysis revealed a significant enrichment of corticosteroids (ATC code H02, FDR-corrected P-value 0.022) including many interacting with glucocorticoid receptor (NR3C1, FDR-corrected P-value 1.5E-5).

CARTʼs network visualization allows the user to quickly explore enrichments of various bioactivities and whether these are functionally coherent, which is illustrated by our analysis of Cluster 10. This enrichment network (Supplementary Figure 4) shows associations between most drugs in this cluster and the histamine receptor H1 as the primary target (FDR-corrected P value 0.0019) consistent with the enrichment of the ATC codes R06/R06A, “Antihistamines for systemic use”, (FDR-corrected P value 0.00062/0.0014), and the ChEMBL functional therapeutic classification “Anti-histamine receptor activity agent” (FTC_A0004969, FDR-corrected P value 0.032). In general, CARTʼs network visualization can also be useful for investigating the effects of polypharmacological agents such as psychotropic drugs that modulate multiple targets or to reveal sub-clusters in the input drug set that are associated with different (off-)targets, side-effects or other bioactivities.

**8. Supplementary Table 1. Comparison of CART’s features to existing tools**

**References**

Croset,S. *et al.* (2014) The functional therapeutic chemical classification system. *Bioinformatics*, **30**, 876–83.

Davis,A.P. *et al.* (2015) The Comparative Toxicogenomics Database’s 10th year anniversary: Update 2015. *Nucleic Acids Res.*, **43**, D914–D920.

Kuhn,M. *et al.* (2014) STITCH 4: integration of protein-chemical interactions with user data. *Nucleic Acids Res.*, **42**, D401–7.

Kuhn,M. *et al.* (2015) The SIDER database of drugs and side effects. *Nucleic Acids Res.*

Law,V. *et al.* (2014) DrugBank 4.0: shedding new light on drug metabolism. *Nucleic Acids Res.*, **42**, D1091–7.

Rihel,J. *et al.* (2010) Zebrafish behavioral profiling links drugs to biological targets and rest/wake regulation. *Science (80-. ).*, **327**, 348.

Zhu,F. *et al.* (2012) Therapeutic target database update 2012: a resource for facilitating target-oriented drug discovery. *Nucleic Acids Res.*, **40**, D1128–36.
